# Feasibility study of veterinary antibiotic consumption in Germany - comparison of ADDs and UDDs by animal production type, antimicrobial class and indication

**DOI:** 10.1186/1746-6148-10-7

**Published:** 2014-01-08

**Authors:** Roswitha Merle, Matthias Robanus, Christine Hegger-Gravenhorst, Yvonne Mollenhauer, Peter Hajek, Annemarie Käsbohrer, Walther Honscha, Lothar Kreienbrock

**Affiliations:** 1 Department of Biometry, Epidemiology and Information Processing, University of Veterinary Medicine Hannover, WHO-Centre Veterinary Public Health, Bünteweg 2, Hannover D-30559, Germany; 2 Veterinary Faculty of Universität Leipzig, Institute for Pharmacology, Pharmacy and Toxicology, An den Tierkliniken 15, Leipzig D-04103, Germany; 3 Department Biological Safety, Federal Institute for Risk Assessment, Max-Dohrn-Str. 8-10, Berlin 10589, Germany

**Keywords:** VetCAb, Livestock, Tetracyclines, Fluoroquinolones, Macrolides, Cephalosporins

## Abstract

**Background:**

Within a feasibility study the use of antibiotics in pigs and cattle was determined in 24 veterinary practices in Lower Saxony and on 66 farms in North Rhine-Westphalia in Germany. Focus was laid on the comparison of the Used Daily Doses (UDD) (dose per animal and day prescribed by the veterinarians) with the Defined Animal Daily Doses (ADD) (dose per animal and day calculated by means of recommended dosages and estimated live weights).

**Results:**

For piglets and calves most of the UDD (50% and 46% of nUDD, respectively) were above the ADD (i.e. UDD/ADD-ratio above 1.25). Regarding sows, fattening pigs, dairy and beef cattle, most of the UDDs (49% to 65% of nUDD) were lower than the respective ADD (i.e. UDD/ADD-ratio below 0.8). In pigs, the UDDs of beta-lactams, fluoroquinolones and cephalosporins, and in cattle, those of macrolides and beta-lactams were often below the ADDs. Tetracyclines were frequently used above the recommended dose.

Enteric diseases were more often treated below the recommended dose than respiratory diseases, possibly due to overestimation of the live weight (diarrhea in young animals, respiratory diseases in elder animals) and consequently overestimation of the recommended dose.

**Conclusion:**

Comparisons between UDD and ADD can be used to observe differences between antimicrobials and trends in the usage of antibiotics. But individual treatment comparisons of UDD and ADD must be interpreted carefully, because they may be due to lower live weights than estimated. Correlating such data with data on the occurrence of resistant bacteria in future may help to improve resistance prevention and control.

## Background

Use of antibiotics causes the risk of selecting for resistant bacteria [1,2]. In order to avoid resistances and to ensure therapeutic efficacy prudent use of antimicrobials is necessary [3]. In Germany, the respective guidelines were published by the working group “veterinary drugs” of the Federal Veterinary Chamber [4].

According to the EFSA Panel on Biological Hazards, resistance monitoring has to be supplemented by the collection of data on the antibiotic use in humans as well as in the veterinary sector [5]. The German Antibiotic Resistance Strategy DART released by the German ministries in 2008 identified the collection of sales and consumption data as one of the measures which should be taken to control antibiotic resistances in human and veterinary medicine [6]. The European Medicines Agency EMA initiated the European Surveillance of Veterinary Antimicrobial Consumption ESVAC, a project which collects sales data from EU member states in a harmonized protocol [7-9]. While this project started in 2005 with 10 countries, participation increased in 2010 to 19 countries [10] and in 2011 to 25 countries [11]. In this project, a decrease in the consumption of antibiotics by 8.2% between 2005 and 2009 was observed, between 2010 and 2011 the decline varied from 0 to 28% in the regularly participating countries. However, the authors stated that this decrease may be due to antimicrobials with high dosages (e.g. tetracyclines) having been replaced by antimicrobials with lower dosages, e.g., fluoroquinolones, cephalosporins, and pleuromutilins. The animal population per country was expressed as population correction units (PCU) which relates the numbers of animals per animal species to the respective live weights. The calculated results in mg per PCU differed widely between the countries in nearly all antimicrobial classes, accounted for in part by different distributions of animal species. Countries with a high percentage of pigs seem to use more mg active ingredients per PCU than others. The impact of tetracyclines (mg/PCU) ranged from 4 to 59% of all mg/PCU (average 39%), while cephalosporins reached an average of 0.5% of all mg/PCU.

Currently, some Scandinavian countries like Denmark [12], Sweden [13], and Norway [14] collect data from pharmacies, distributors and the pharmaceutical industry, which reflect the amount of antimicrobials used in the individual animal populations. In contrast to this, in Germany, within a scientific study [15] data were collected on the amount used by veterinarians as well as the number of treatments. This was possible, as veterinarians sell antimicrobials directly to the farmer, and detailed records are kept on this. In other European countries veterinarians distribute drugs directly as well, e.g., in France, Austria, the United Kingdom and in Switzerland [16-20]. In Austria, sales data are collected on a mandatory basis, but data on antibiotic use are evaluated completely only for poultry, and on study basis for cattle and pigs [9,20]. In the Netherlands, the situation is similar to that in Austria with complete sales data, complete consumption data in poultry and study data for pigs and cattle [21]. In Switzerland, complete data on sales and study data on consumption are available as well [10,11,16,20].

Denmark has very strict regulations regarding the documentation of antibiotic use in livestock. Veterinarians have to enter all cases of application of antibiotics into a database. The degree to which prescription data are currently used for quantification of antimicrobial usage is varying between these countries.

Detailed data on the use of antibiotics in farm animals – e.g., by species, age and number of animals, indication of treatment and treatment duration – can only be collected and evaluated when respective farm characteristics as well as prescription details are known.

Three general concepts are available for analyzing the data and quantifying the antibiotic use. First, the amount of active ingredients per se can be used. This variable is precise, but must be regarded separately for each antimicrobial [22] and depends very much on the population size and type of production considered. Secondly, the frequency of applications, e.g., the number of Used Daily Doses (nUDD), can be calculated, if detailed application data are available [23]. The third option is to estimate an application frequency by sales or usage data, if application details are not available. Several concepts from different countries exist for calculating this third option, e.g., Defined Animal Daily Doses (ADD) in Denmark [12,22] and in the Netherlands [21].

Usually, this type of data is used for estimating the number of Defined Animal Daily Doses (nADD) by dividing the amount per antimicrobial used by the previously fixed Animal Daily Dose (ADD), which is the product of expected dosage and the average animal weight. If more detailed data are collected, the number of Used Daily Doses (nUDD = number of animals treated multiplied by the treatment days and the number of active ingredients in the product) or the number of Prescribed Daily Dose (nPDD) per livestock unit and per ATCVetCode can be calculated [24].

One study from Belgium compared nUDD and nADD regarding treatment incidences and showed only slight differences [25]. Other studies also use UDD and ADD for dosage comparisons. Regula et al. investigated prescription patterns of antimicrobials in veterinary practices in Switzerland and observed that most of the prescriptions were dosed within the recommended range in cattle and calves as well as in fattening pigs [26]. Overdosing was seen in adult pigs and dogs. Dosage patterns differ between antimicrobial classes with common dosage above the recommended range for tetracyclines, and underdosing for sulfonamides, aminoglycosides and quinolones. González et al. [27] found similar results on Swiss dairy farms with 30 – 55% underdosing depending on antimicrobial classes and overdosing of cephalosporins, sulfonamides & trimethoprim and macrolides. Pardon et al. [28] reported underdosing in 43.7% of treatments in veal calves. In a study of 50 pig herds in Belgium, the authors stated that parenteral treatment was generally overdosed, while oral applications were often underdosed [29].

In order to evaluate the preconditions for a German monitoring system of veterinary antibiotic use, a feasibility study “VetCAb” (Veterinary consumption of antibiotics) was carried out that collected consumption data in a bottom-up approach [15]. Parts of the study that concern the UDD per animal and year as well as the administration routes have already been published [15].

The aim of the analysis presented here focuses on comparing the number of UDDs with the number of ADDs by the UDD/ADD-ratio per antimicrobial class and to discuss the impact of the type of indicator calculated on the interpretation of the usage patterns by animal groups. For this purpose, also UDD/ADD-ratios for administration routes and indications were calculated separately. As on EU level only data on nADDs might be available in the future [30], it is discussed whether the calculating nADD has major pitfalls for assessing of the antibiotic use in livestock.

## Methods

Data from the feasibility study in Germany, already previously described [15] were used for this additional analysis. Briefly, in the study were 24 veterinary surgeries as well as 66 livestock farms involved. All applications of antibiotics of a one-year-period (September 1, 2006 – August 31, 2007) were assessed [15]. The data sources were the veterinarians’ official application records following article 13 of the Regulation of Veterinary Pharmacies [31]. These documents contain information on animal species, age and number of animals, the trade name of the pharmaceutical drug and the amount (ml, g or injectors) as well as the indication, pharmaceutical form, and duration of the treatment for every drug administered separately.

In order to validate the identity and amount of the administered drugs relevant data from the system of the Veterinary Information Service for Drug Use, Toxicology and Pharmaceutical Legislation VETIDATA (http://www.vetidata.de) were made available to the study database for background information on the drugs.

All analyses were carried out for the individual antimicrobials and antimicrobial classes. For each record the administered or prescribed amount of active ingredient was calculated. The number of used daily doses nUDD was defined as the number of animals treated multiplied by the treatment days (both noted in the record) and by the number of active ingredients in the product. The Used Daily Dose UDD was calculated as g active ingredients per animal and day for each antimicrobial per record (amount active ingredients per antimicrobial divided by number of animals treated and treatment days). The number of Defined Animal Daily Doses nADD was calculated according to Merle et al. [15] and Jensen et al. [22]. In short, the Defined Animal Daily Dose ADD for each antimicrobial and production type was calculated by multiplying the recommended dosage for this antimicrobial and the standard weight for the species, weight group/production type for the respective animal production type. Dividing the amount of active ingredients per record by this ADD resulted in the nADD. The standard weight for the animal species, weight group/production type was derived from estimated live weights at treatment of livestock as defined according to Merle et al. [15] and MARAN 2007 [32] as follows: sows 220 kg, suckling piglets 12.5 kg, weaners 25 kg, finishers 70.2 kg, cows and bulls 600 kg, calves 80 kg. The recommended dosage (mg active substance/kg live weight and day) was defined for both parenteral and oral application separately for the species cattle and pigs on the basis of the Summary of Product Characteristics (SPC) and in some cases are based on information from the scientific literature and expert opinions as laid down in the VETIDATA system.

In order to compare usage patterns for different animal groups, antimicrobials, administration routes and indications, for each record the UDD was divided by the ADD to obtain the UDD/ADD-ratio per record. Ratios between 0.8 and 1.25 were regarded as appropriate. Ratios under 0.8 were regarded as “below recommended dose” and those ratios above 1.25 were denoted as “above recommended dose”. In the tables, results are displayed as percentages of records and of nUDDs that were below, within or above the respective recommended dose.

46,201 complete records from pigs and cattle were considered for analysis. Locally administered antibiotics as well as incomplete records were excluded. For analyses per antimicrobial, the antimicrobials of preparations with two or three active ingredients were regarded separately, leading to 58,923 antimicrobial-related data set entries.

Descriptive statistical analyses, analyses of variance as well as chi-square-statistics were carried out using SAS®, Version 9.2 TS Level 1 M3 (SAS Institute Inc., Cary, NC, USA). In order to compare the differences between the percentage of records and the respective percentage of nUDD per dose category, analyses of variance were carried out.

## Results

In total, 57% of all records were related to pigs, resulting in 96% of all nUDD and 94% of all nADD. Data for piglets, sows and fattening pigs are summarized in Tables 1, 2 and 3.

**Table 1:** Percentages of records and nUDDs with doses below, within and above the recommended doses in piglets in a feasibility study of antibiotic use in Germany including 19 veterinary practices and 26 farms

	**Records**	**%**			**Below**		**Within**		**Above**	
			**nUDD**	**%**	**% Records**	**% nUDD**	**% Records**	**% nUDD**	**% Records**	**% nUDD**
**Macrolides**	984	7.7	1599436	12.2	24.7	51.2	23.0	18.2	52.3	30.6
**Beta-Lactams**	4879	38.3	2942528	22.4	38.6	32.1	15.9	15.6	45.5	52.2
**Aminoglycosides**	725	5.7	308031	2.3	55.6	26.1	7.6	3.6	36.8	70.3
**Fenicoles**	158	1.2	18798	0.1	1.9	14.0	26.6	30.9	71.5	55.1
**Tetracyclines**	1841	14.4	2943293	22.4	25.1	26.0	21.2	27.5	53.7	46.4
**Lincosamides**	542	4.2	478013	3.6	40.8	56.8	38.9	15.7	20.3	27.5
**Polypeptides**	1285	10.1	1942674	14.8	24.6	20.2	20.2	19.1	55.3	60.7
**Sulfonamides**	707	5.5	2222599	16.9	27.6	15.6	25.2	21.5	47.2	62.9
**Fluoroquinolones**	882	6.9	144637	1.1	26.0	41.0	18.5	23.2	55.6	35.8
**Cephalosporins**	497	3.9	143417	1.1	23.5	39.4	5.4	4.8	71.0	55.8
**Pleuromutilines**	255	2.0	384482	2.9	46.3	57.6	29.4	26.7	24.3	15.7
**All**	12755	100.0	13127908	100.0	32.9	30.2	18.8	20.2	48.3	49.7

**Table 2:** Percentages of records and nUDDs with doses below, within and above the recommended doses in sows in a feasibility study of antibiotic use in Germany including 19 veterinary practices and 26 farms

	**Records**	**%**			**Below**		**Within**		**Above**	
			**nUDD**	**%**	**% Records**	**% nUDD**	**% Records**	**% nUDD**	**% Records**	**% nUDD**
**Macrolides**	163	2.8	24581	3.4	43.6	82.7	33.7	14.5	22.7	2.8
**Beta-Lactams**	1832	31.4	159867	22.3	36.7	82.2	26.3	10.4	37.0	7.3
**Aminoglycosides**	359	6.2	7658	1.1	55.7	83.4	8.9	4.3	35.4	12.3
**Fenicoles**	78	1.3	1074	0.1	17.9	39.9	46.2	45.3	35.9	14.9
**Tetracyclines**	617	10.6	340661	47.5	32.4	56.9	21.9	17.3	45.7	25.9
**Lincosamides**	180	3.1	17671	2.5	71.1	94.8	15.6	2.4	13.3	2.8
**Polypeptides**	162	2.8	39314	5.5	84.6	75.5	10.5	12.8	4.9	11.8
**Sulfonamides**	738	12.7	86593	12.1	80.1	52.3	7.6	21.0	12.3	26.7
**Fluoroquinolones**	1131	19.4	21185	3.0	34.5	60.0	20.7	16.0	44.8	24.0
**Cephalosporins**	555	9.5	11257	1.6	35.9	78.1	16.8	9.3	47.4	12.7
**Pleuromutilines**	17	0.3	7314	1.0	35.3	3.0	58.8	97.0	5.9	0.0
**All**	5832	100.0	717175	100.0	44.7	64.9	20.2	16.0	35.1	19.0

**Table 3:** Percentages of records and nUDDs with doses below, within and above the recommended doses in fattening pigs in a feasibility study of antibiotic use in Germany including 19 veterinary practices and 26 farms

	**Records**	**%**			**Below**		**Within**		**Above**	
			**nUDD**	**%**	**% Records**	**% nUDD**	**% Records**	**% nUDD**	**% Records**	**% nUDD**
**Macrolides**	1447	9.7	2107065	16.3	46.1	75.9	27.5	18.3	26.4	5.8
**Beta-Lactams**	4862	32.6	2906106	22.5	37.1	41.3	19.9	29.2	43.0	29.6
**Aminoglycosides**	626	4.2	157925	1.2	88.2	55.0	8.8	34.1	3.0	10.9
**Fenicoles**	491	3.3	22549	0.2	7.1	18.9	38.5	43.5	54.4	37.5
**Tetracyclines**	2814	18.9	3823986	29.6	33.9	44.3	25.3	31.0	40.7	24.7
**Lincosamides**	1032	6.9	564908	4.4	46.0	57.9	28.2	28.4	25.8	13.7
**Polypeptides**	593	4.0	1116686	8.6	34.9	34.3	26.1	29.6	39.0	36.1
**Sulfonamides**	749	5.0	1663887	12.9	54.6	38.2	18.3	29.8	27.1	32.0
**Fluoroquinolones**	1414	9.5	93777	0.7	14.9	41.4	28.3	19.6	56.8	39.0
**Cephalosporins**	466	3.1	42230	0.3	36.5	74.1	25.5	15.4	38.0	10.6
**Pleuromutilines**	424	2.8	428155	3.3	71.7	78.3	21.7	17.8	6.6	3.9
**All**	14918	100.0	12927274	100.0	38.8	49.0	23.6	27.6	37.6	23.4

In piglets, 22% of all records accounted for 47% of all nUDD and 58% of all nADD. Beta-lactams and tetracyclines were used most often (nUDD) followed by sulfonamides & trimethoprim (Table 1). 48% of the records and 50% of nUDD were above the recommended dose (i.e. UDD/ADD-ratio above 1.25), and only 19% of the records (20% of nUDD) were within the recommended dose (i.e. UDD/ADD-ratio between 0.8 and 1.25). Tetracyclines and polypeptides were more often above the recommended dose than within or below the recommended dose.

10% of all records, but only 2.6% of all nUDD and 1.7% of nADD were linked to sows (Table 2). All antimicrobial classes were used to some extent below the recommended dose, but 35% of the records showed doses above the recommended dose. Differences between the percentage of records and the percentage of nUDD were statistically significant (analysis of variance, p-value 0.0038).

Fattening pigs accounted for the highest number of records (25%) and of nUDD (46%) (34% of nADD)(Table 3). Apart from sulfonamides & trimethoprim, fenicoles, and polypeptides, all antimicrobials were used below the recommended dose. 39% of the records were below the recommended dose (49% of nUDD), 38% above (23% of nUDD) (statistically significant differences between% of records and% of nUDD in the analysis of variance, p-value 0.0167).

14% of all records, but only 4% of all nUDD and 6% of nADD related to calves (Table 4). Calves often received higher doses than recommended. The frequently used antimicrobial classes tetracyclines and sulfonamides & trimethoprim had 63% and 46% of nUDD above the recommended dose, respectively.

**Table 4:** Percentages of records and nUDDs with doses below, within and above the recommended doses in calves in a feasibility study of antibiotic use in Germany including 19 veterinary practices and 26 farms

	**Records**	**%**			**Below**		**Within**		**Above**	
			**nUDD**	**%**	**% Records**	**% nUDD**	**% Records**	**% nUDD**	**% Records**	**% nUDD**
**Macrolides**	811	9.6	30229	2.7	9.4	8.2	38.5	27.9	52.2	63.9
**Beta-Lactams**	2176	25.7	146041	13.3	47.6	41.4	23.0	22.0	29.4	36.6
**Aminoglycosides**	1023	12.1	90515	8.2	55.7	61.2	29.2	30.7	15.1	8.1
**Fenicoles**	636	7.5	10934	1.0	46.9	73.8	27.2	15.8	25.9	10.4
**Tetracyclines**	892	10.5	333302	30.3	17.7	24.0	15.4	13.5	66.9	62.5
**Polypeptides**	231	2.7	44368	4.0	45.9	15.4	22.5	52.4	31.6	32.2
**Sulfonamides**	566	6.7	411779	37.5	28.1	23.6	22.4	30.3	49.5	46.1
**Fluoroquinolones**	1805	21.3	27814	2.5	31.6	63.7	24.4	13.6	44.0	22.7
**Cephalosporins**	339	4.0	4536	0.4	12.4	38.0	26.3	12.5	61.4	49.5
**All**	8479	100.0	1099518	100.0	35.6	30.0	25.1	24.3	39.3	45.7

1.7% of records, 0.1% of nUDD and 0.05% of nADD were linked to dairy cattle. Beta-lactams, cephalosporins and sulfonamides & trimethoprim accounted for the highest numbers of nUDD (Table 5). 44% of the records were below the recommended dose, but 28% above (analysis of variance, p-value 0.0238). Only tetracyclines were often above the recommended dose. Cephalosporins and fluoroquinolones were less frequently below the recommended dose than the other antimicrobials.

**Table 5:** Percentages of records and nUDDs with doses below, within and above the recommended doses in dairy cattle in a feasibility study of antibiotic use in Germany including 19 veterinary practices and 26 farms

	**Records**	**%**			**Below**		**Within**		**Above**	
			**nUDD**	**%**	**% Records**	**% nUDD**	**% Records**	**% nUDD**	**% Records**	**% nUDD**
**Macrolides**	824	5.2	4538	5.3	35.1	60.8	49.4	33.5	15.5	5.7
**Beta-Lactams**	4737	29.7	22766	26.5	46.7	64.6	26.3	20.2	26.9	15.2
**Aminoglycosides**	1291	8.1	5300	6.2	68.2	70.4	17.3	20.0	14.6	9.6
**Fenicoles**	81	0.5	714	0.8	86.4	96.2	12.3	3.6	1.2	0.1
**Tetracyclines**	555	3.5	6243	7.3	42.7	36.1	9.5	12.2	47.7	51.7
**Polypeptides**	510	3.2	2366	2.8	58.6	83.8	19.0	10.5	22.4	5.7
**Sulfonamides**	1209	7.6	15926	18.5	88.5	68.4	3.5	20.1	8.0	11.5
**Fluoroquinolones**	2713	17.0	11780	13.7	21.9	55.5	36.7	20.7	41.4	23.8
**Cephalosporins**	4009	25.2	16309	19.0	33.8	36.0	35.9	37.7	30.3	26.3
**All**	15929	100.0	85942	100.0	44.0	57.5	28.4	23.3	27.6	19.2

Only 2% of all records and 0.1% of all nUDD as well as of all nADD were related to beef cattle (Table 6). For most antimicrobial classes treatments were often below the recommended dose, but treatments with tetracyclines, fluoroquinolones and cephalosporins were rarely below the recommended dose.

**Table 6:** Percentages of records and nUDDs with doses below, within and above the recommended doses in beef cattle in a feasibility study of antibiotic use in Germany including 19 veterinary practices and 26 farms

	**Records**	**%**			**Below**		**Within**		**Above**	
			**nUDD**	**%**	**% Records**	**% nUDD**	**% Records**	**% nUDD**	**% Records**	**% nUDD**
**Macrolides**	141	14.0	1132	6.0	57.4	56.9	35.5	37.1	7.1	6.0
**Beta-Lactams**	465	46.0	4698	25.0	71.6	90.3	18.1	7.2	10.3	2.5
**Aminoglycosides**	17	1.7	86	0.5	52.9	57.0	23.5	32.6	23.5	10.5
**Fenicoles**	114	11.3	1184	6.3	92.1	98.4	6.1	1.3	1.8	0.3
**Tetracyclines**	56	5.5	6233	33.1	50.0	15.6	12.5	6.9	37.5	77.5
**Polypeptides**	2	0.2	49	0.3	100.0	100.0	.	.	.	.
**Sulfonamides**	16	1.6	4586	24.4	68.8	85.0	18.8	12.0	12.5	3.1
**Fluoroquinolones**	111	11.0	479	2.5	27.9	22.1	20.7	22.8	51.4	55.1
**Cephalosporins**	88	8.7	380	2.0	33.0	28.7	31.8	36.8	35.2	34.5
**All**	1010	100.0	18827	100.0	62.3	59.7	20.4	10.8	17.3	29.6

The majority of nUDD were administered orally to pigs as well as to cattle (Table 7). In pigs, the distribution of records and nUDD regarding the dose categories differed only slightly between parenteral and oral applications. Only in fattening pigs were oral applications more frequently below the recommended dose than parenteral applications (chi-square-test, p-value < 0.0001).

**Table 7:** Percentages of records and nUDDs with doses below, within and above the recommended doses per administration route and animal group in a feasibility study of antibiotic use in Germany including 19 veterinary practices and 26 farms

		**Records**	**%**			**Below**		**Within**		**Above**	
				**nUDD**	**%**	**% Records**	**% nUDD**	**% Records**	**% nUDD**	**% Records**	**% nUDD**
**Piglets**	**Parenteral**	7090	55.6	1366875	10.4	34.9	47.8	17.8	16.2	47.3	36.0
	**Oral**	5665	44.4	11761033	89.6	30.3	28.1	20.1	20.6	49.6	51.3
	**All**	12755	100.0	13127908	100.0	32.9	30.2	18.8	20.2	48.3	49.7
**Sows**	**Parenteral**	5143	88.2	112166	15.6	44.9	74.9	20.0	11.9	35.1	13.3
	**Oral**	689	11.8	605009	84.4	43.1	63.1	21.6	16.8	35.3	20.1
	**All**	5832	100.0	717175	100.0	44.7	64.9	20.2	16.0	35.1	19.0
**Fattening Pigs**	**Parenteral**	8912	59.7	626469	4.8	35.0	62.5	21.4	15.1	43.6	22.4
	**Oral**	6006	40.3	12300805	95.2	44.4	48.3	26.8	28.2	28.8	23.4
	**All**	14918	100.0	12927274	100.0	38.8	49.0	23.6	27.6	37.6	23.4
**Calves**	**Parenteral**	6573	77.5	120400	11.0	37.4	69.7	27.1	15.1	35.5	15.2
	**Oral**	1906	22.5	979118	89.0	29.2	25.1	18.2	25.5	52.6	49.4
	**All**	8479	100.0	1099518	100.0	35.6	30.0	25.1	24.3	39.3	45.7
**Dairy Cattle**	**Parenteral**	15851	99.5	69855	81.3	44.0	58.5	28.4	23.6	27.6	17.9
	**Oral**	78	0.5	16087	18.7	44.9	53.2	16.7	22.1	38.5	24.7
	**All**	15929	100.0	85942	100.0	44.0	57.5	28.4	23.3	27.6	19.2
**Beef Cattle**	**Parenteral**	966	95.6	6657	35.4	63.4	75.6	20.4	15.3	16.3	9.1
	**Oral**	44	4.4	12170	64.6	38.6	51.0	20.5	8.3	40.9	40.7
	**All**	1010	100.0	18827	100.0	62.3	59.7	20.4	10.8	17.3	29.6

In cattle, oral applications were more often above the recommended dose, whereas most of the doses of parenteral applications were below the recommended dose. Differences regarding the dose category between oral and parenteral applications (records) were statistically significant (chi-square-test, p-value < 0.0001).

Predominant indications for antimicrobial agents used in this study were respiratory diseases, as intestinal diseases (Table 8). Apart from sows and dairy cattle, the percentage of records below the recommended dose was higher regarding enteric diseases than respiratory diseases (chi-square-test, p-value < 0.0001 for piglets, fattening pigs and calves).

**Table 8:** Percentages of records and nUDDs with doses below, within and above the recommended doses per indication and animal group in a feasibility study of antibiotic use in Germany including 19 veterinary practices and 26 farms

		**Records**	**%**			**Below**		**Within**		**Above**	
				**nUDD**	**%**	**% Records**	**% nUDD**	**% Records**	**% nUDD**	**% Records**	**% nUDD**
**Piglets**	**Respiratory disease**	5093	39.9	5904075	45.0	24.1	18.2	23.7	24.1	52.2	57.6
	**Skin disease**	303	2.4	106938	0.8	48.2	30.7	21.5	19.8	30.4	49.5
	**Enteric disease**	2914	22.8	4154307	31.6	33.5	33.8	18.1	18.9	48.4	47.3
	**Articular disease**	688	5.4	113860	0.9	76.0	80.7	6.7	6.3	17.3	13.1
	**CNS disease**	11	0.1	2554	0.0	18.2	78.3	18.2	7.9	63.6	13.8
	**Reproductive system disease**	22	0.2	49900	0.4	40.9	72.9	36.4	16.6	22.7	10.5
	**Other**	3722	29.2	2796054	21.3	35.1	47.1	14.6	14.3	50.3	38.6
	**All**	12753	100.0	13127688	100.0	32.9	30.2	18.8	20.2	48.3	49.7
**Sows**	**Respiratory disease**	991	17.0	175959	24.5	57.0	73.6	20.2	18.0	22.8	8.3
	**Skin disease**	34	0.6	526	0.1	47.1	42.8	14.7	35.9	38.2	21.3
	**Enteric disease**	171	2.9	77547	10.8	50.3	67.2	21.6	19.7	28.1	13.0
	**Articular disease**	161	2.8	1907	0.3	32.3	62.6	24.2	15.5	43.5	21.9
	**CNS disease**	1	0.0	3	0.0	.	.	100.0	100.0	.	.
	**Udder disease**	66	1.1	377	0.1	39.4	45.4	21.2	15.1	39.4	39.5
	**Reproductive system disease**	2428	41.6	69830	9.7	47.9	57.8	11.5	12.7	40.6	29.5
	**Other**	1980	34.0	391026	54.5	35.3	61.9	30.5	15.0	34.2	23.1
	**All**	5832	100.0	717175	100.0	44.7	64.9	20.2	16.0	35.1	19.0
**Fattening pigs**	**Respiratory disease**	8273	55.5	6138179	47.5	37.3	42.0	23.9	29.0	38.8	29.0
	**Skin disease**	643	4.3	52603	0.4	29.5	66.8	11.8	19.9	58.6	13.2
	**Enteric disease**	2539	17.0	4931365	38.2	50.6	56.2	26.9	27.1	22.4	16.7
	**Articular disease**	511	3.4	15563	0.1	38.4	77.7	15.5	8.6	46.2	13.6
	**CNS disease**	2	0.0	80	0.0	100.0	100.0	.	.	.	.
	**Reproductive system disease**	66	0.4	51465	0.4	43.9	95.1	21.2	3.5	34.8	1.4
	**Other**	2881	19.3	1731489	13.4	34.5	50.9	23.8	25.5	41.7	23.6
	**All**	14915	100.0	12920744	100.0	38.8	49.0	23.6	27.6	37.6	23.4
**Calves**	**Respiratory disease**	4624	54.6	794350	72.2	27.2	26.5	26.1	22.6	46.7	50.9
	**Skin disease**	92	1.1	490	0.0	44.6	32.7	12.0	11.8	43.5	55.5
	**Enteric disease**	1586	18.7	175228	15.9	51.5	48.1	21.1	27.6	27.4	24.3
	**Articular disease**	216	2.6	3528	0.3	64.8	83.1	18.5	10.3	16.7	6.5
	**CNS disease**	10	0.1	25	0.0	30.0	12.0	10.0	4.0	60.0	84.0
	**Reproductive system disease**	1	0.0	2	0.0	.	.	.	.	100.0	100.0
	**Other**	1936	22.9	125851	11.4	39.0	25.2	27.7	31.0	33.3	43.8
	**All**	8465	100.0	1099474	100.0	35.6	30.0	25.1	24.3	39.3	45.7
**Dairy cattle**	**Respiratory disease**	1497	9.4	20123	23.4	60.8	64.4	18.8	21.3	20.4	14.3
	**Skin disease**	893	5.6	3840	4.5	27.1	30.2	34.9	38.8	38.0	31.0
	**Enteric disease**	202	1.3	2678	3.1	54.5	81.1	26.7	8.4	18.8	10.5
	**Articular disease**	728	4.6	3115	3.6	27.2	32.5	38.2	41.4	34.6	26.0
	**CNS disease**	4	0.0	4	0.0	50.0	50.0	25.0	25.0	25.0	25.0
	**Udder disease**	6394	40.1	21296	24.8	38.2	45.8	31.2	31.0	30.6	23.2
	**Reproductive system disease**	1386	8.7	5214	6.1	55.0	60.2	19.4	16.8	25.6	23.0
	**Other**	4825	30.3	29672	34.5	48.5	64.8	27.5	17.6	23.9	17.5
	**All**	15929	100.0	85942	100.0	44.0	57.5	28.4	23.3	27.6	19.2
**Beef cattle**	**Respiratory disease**	489	48.4	12270	65.2	64.8	54.3	18.0	10.4	17.2	35.2
	**Skin disease**	55	5.4	201	1.1	41.8	32.8	40.0	46.8	18.2	20.4
	**Enteric disease**	13	1.3	1590	8.4	38.5	93.4	30.8	5.9	30.8	0.7
	**Articular disease**	158	15.6	1333	7.1	74.1	89.3	16.5	8.6	9.5	2.1
	**Reproductive system disease**	4	0.4	4	0.0	75.0	75.0	25.0	25.0	.	.
	**Other**	291	28.8	3429	18.2	56.4	53.2	22.3	12.9	21.3	33.9
	**All**	1010	100.0	18827	100.0	100.0	59.7	20.4	10.8	17.3	29.6

Tables 9 and 10 summarize the distribution of the used dosages and the respective recommended dosage per antimicrobial and per administration route for pigs and cattle respectively. For pigs and cattle differently, for a few antimicrobials the used dosages were frequently below the respective recommended dosage. In pigs, only spectinomycin and pleuromutilines showed lower dosages than recommended, whereas oxytetracycline was frequently used above the recommended dosage. In cattle, tetracycline was frequently used above the recommended dosage, while for ampicillin, spectinomycin, and sulfonamides & trimethoprim used dosages were below the recommended dosage.

**Table 9:** Used and recommended dosages per antimicrobial and administration route in pigs in a feasibility study of antibiotic use in Germany including 19 veterinary practices and 26 farms

			**Used dosage**				**Recommended dosage**
			**Records**	**25%-Quartile**	**Median**	**75%-Quartile**	
**Macrolides**	**Acetylisovaleryltylosin**	**Oral**	2	3.7	3.7	3.7	3.0
	**Erythromycin**	**Parenteral**	17	11.1	13.3	16.7	22.0
	**Tilmicosin**	**Oral**	110	7.1	12.3	19.0	20.0
	**Tulathromycin**	**Parenteral**	547	2.0	4.0	8.0	2.5
	**Tylosin**	**Parenteral**	749	12.5	17.5	24.0	15.0
		**Oral**	1169	8.5	12.1	19.2	17.0
**Beta-Lactames**	**Amoxicillin**	**Parenteral**	2249	13.9	18.5	30.0	10.0
		**Oral**	2792	20.0	34.8	57.1	30.0
	**Ampicillin**	**Parenteral**	453	8.0	12.1	20.0	37.0
		**Oral**	11	0.3	11.2	31.6	45.0
	**Benzylpenicillin-Procaine**	**Parenteral**	4407	12.0	22.9	36.0	20.0
	**Benzylpenicillin-Benzathine**	**Parenteral**	1661	5.6	7.6	14.5	20.0
**Aminoglycosides**	**Apramycin**	**Oral**	103	3.4	5.6	16.9	15.0
	**Gentamicin**	**Parenteral**	332	3.3	5.6	10.2	6.0
	**Kanamycin**	**Parenteral**	2	6.9	13.5	20.0	15.0
	**Neomycin**	**Parenteral**	209	4.9	11.7	29.2	7.0
		**Oral**	164	8.5	14.0	14.0	7.0
	**Spectinomycin**	**Parenteral**	900	9.1	13.0	19.6	30.0
**Fenicoles**	**Florfenicol**	**Parenteral**	727	15.0	20.0	30.0	15.0
**Tetracyclines**	**Chlortetracycline**	**Oral**	1062	18.6	33.2	73.7	40.0
	**Doxycycline**	**Oral**	35	7.9	11.1	20.0	15.0
	**Oxytetracycline**	**Parenteral**	1365	16.7	25.0	36.0	15.0
		**Oral**	281	22.9	34.3	53.3	80.0
	**Tetracycline**	**Oral**	2529	38.5	56.6	77.6	50.0
**Lincosamides**	**Lincomycin**	**Parenteral**	1269	5.0	9.8	11.1	10.0
		**Oral**	485	3.8	6.5	12.4	10.0
**Polypeptides**	**Colistin**	**Parenteral**	368	0.8	1.5	2.5	2.5
		**Oral**	1672	3.8	6.9	13.9	5.0
**Sulfonamides**	**Sulfadimidine**	**Oral**	425	15.0	25.0	80.0	75.0
	**Sulfamethoxpyridazine**	**Parenteral**	5	57.9	57.9	57.9	60.0
**Fluoroquinolones**	**Danofloxacin**	**Parenteral**	846	1.7	2.1	2.5	1.3
	**Enrofloxacin**	**Parenteral**	1419	2.8	4.0	6.0	3.5
		**Oral**	140	1.0	1.1	1.7	1.7
	**Marbofloxacin**	**Parenteral**	1022	1.9	2.4	3.7	2.0
**Cephalosporins**	**Cefquinome**	**Parenteral**	1143	1.1	1.7	2.5	1.5
	**Ceftiofur**	**Parenteral**	375	2.0	4.0	6.7	3.0
**Pleuromutilines**	**Tiamulin**	**Parenteral**	214	8.0	11.1	16.0	15.0
		**Oral**	477	6.8	10.1	13.5	15.0
	**Valnemulin**	**Oral**	5	0.6	0.7	0.9	9.0
**Sulfonamides & trimethoprim**	**Sulfadiazine**	**Oral**	877	25.6	34.3	47.6	25.0
	**Sulfadimethoxine**	**Oral**	13	31.6	42.9	150.0	17.0
	**Sulfadimidine**	**Parenteral**	796	19.2	30.0	44.4	75.0
		**Oral**	5	5.5	9.6	32.7	75.0
	**Sulfadoxine**	**Parenteral**	70	10.0	17.8	26.7	16.0
		**Oral**	3	9.6	12.1	50.7	16.0

**Table 10:** Used and recommended dosage per antimicrobial and administration route in cattle in a feasibility study of antibiotic use in Germany including 19 veterinary practices and 26 farms

			**Used dosage**				**Recommended dosage**
			**Records**	**25%-Quartile**	**Median**	**75%-Quartile**	
**Macrolides**	**Erythromycin**	**Parenteral**	142	10.0	10.0	13.3	11.0
	**Tilmicosin**	**Parenteral**	171	7.5	10.9	15.0	10.0
		**Oral**	11	28.1	31.6	37.5	20.0
	**Tulathromycin**	**Parenteral**	878	2.0	2.5	4.0	2.5
	**Tylosin**	**Parenteral**	561	10.0	13.3	14.2	15.0
		**Oral**	13	19.4	33.9	56.0	17.0
**Beta-Lactames**	**Amoxicillin**	**Parenteral**	1648	7.5	10.0	16.0	10.0
		**Oral**	370	13.5	25.0	44.4	30.0
	**Ampicillin**	**Parenteral**	452	7.6	10.0	13.6	37.0
		**Oral**	3	18.8	25.0	25.0	45.0
	**Benzylpenicillin-Procaine**	**Parenteral**	3027	10.0	15.0	24.0	20.0
	**Benzylpenicillin**	**Parenteral**	7	18.0	18.0	18.0	20.0
	**Benzylpenicillin-Benzathine**	**Parenteral**	846	4.8	9.1	11.9	20.0
	**Penethamathydrojodid**	**Parenteral**	1025	9.6	19.1	19.1	10.0
**Aminoglycosides**	**Gentamicin**	**Parenteral**	778	3.3	5.1	6.4	6.0
	**Kanamycin**	**Parenteral**	3	5.0	5.0	10.0	15.0
	**Neomycin**	**Parenteral**	524	5.3	7.0	13.1	7.0
		**Oral**	52	4.1	5.6	10.5	7.0
	**Spectinomycin**	**Parenteral**	974	5.9	9.8	12.5	20.0
**Fenicoles**	**Florfenicol**	**Parenteral**	831	15.0	20.4	37.5	30.0
**Tetracyclines**	**Chlortetracycline**	**Oral**	426	35.3	60.3	92.9	40.0
	**Oxytetracycline**	**Parenteral**	666	10.0	17.3	20.0	15.0
		**Oral**	58	22.1	34.5	41.7	80.0
	**Tetracycline**	**Oral**	353	41.3	66.0	82.5	20.0
**Polypeptides**	**Colistin**	**Parenteral**	622	1.0	1.3	2.6	2.5
		**Oral**	121	5.1	7.1	14.3	5.0
**Sulfonamides**	**Sulfadimidine**	**Oral**	31	34.7	78.1	110.4	75.0
	**Sulfamethoxpyridazine**	**Parenteral**	3	60.0	62.5	62.5	60.0
**Fluoroquinolones**	**Danofloxacin**	**Parenteral**	587	1.7	2.3	4.5	1.3
	**Difloxacin**	**Parenteral**	102	5.2	6.9	10.4	2.5
	**Enrofloxacin**	**Parenteral**	2600	2.5	3.8	5.2	3.5
		**Oral**	29	1.6	3.1	3.5	3.5
	**Marbofloxacin**	**Parenteral**	1208	1.3	2.4	3.1	2.0
		**Oral**	103	0.6	0.6	0.6	2.0
**Cephalosporins**	**Cefquinome**	**Parenteral**	2283	1.2	1.3	1.9	1.5
	**Ceftiofur**	**Parenteral**	2153	1.4	1.7	2.0	1.5
**Sulfonamides & Trimethoprim**	**Sulfadiazine**	**Oral**	405	21.9	33.5	55.8	25.0
	**Sulfadimethoxine**	**Oral**	52	15.0	37.5	60.9	17.0
	**Sulfadimidine**	**Parenteral**	988	24.0	28.8	28.8	75.0
		**Oral**	1	45.0	45.0	45.0	75.0
	**Sulfadoxine**	**Parenteral**	311	12.0	12.0	24.0	16.0

## Discussion

In the present study, it was possible to determine the nUDD, nADD as well as the UDD and the UDD/ADD-ratio on the basis of an ADD. This complements farm related data, calculated per animal under risk, which are published by Merle et al. [15].

The proportions of the antimicrobial classes applied or prescribed in our study were similar to those reported in other countries and with sales data [33]. Tetracyclines, sulfonamides and beta-lactam-antibiotics are used frequently all over Europe [12-14].

To analyze the exposure of animals with antimicrobials and the related selection pressure, the number of individual applications of antimicrobials is more useful information than the amount of antimicrobials used. This is especially true for estimating the impact on antimicrobial resistance selection, as this depends very much on the frequency and length of selection pressure [2]. Therefore, the nUDD displays the frequency of use much better and should be utilized when comparing usage of different antimicrobials and antimicrobial classes.

Other European countries that cannot calculate the nUDD due to lack of detailed data, approximate the number of applications by the Defined Animal Daily Doses ADD which is calculated by the amount of active ingredients, estimated standard live weights and recommended dosages [10,11,21]. As far as the population at risk and the amount of active ingredients per antimicrobial used in the individual animal population is known, the number of standardized doses per animal or per kg live weight can be calculated. By this means, data from different countries, regions or time periods can be compared directly. Thus, this variable is recommended by EMA for monitoring purposes [8].

Trends over time, between antimicrobial classes or animal species can be observed easily by the nUDD as well as the nADD. While the nUDD is evaluated by the veterinarians’ records and thus pictures the real number of used daily doses, the nADD displays an estimate of the number of daily doses. The major drawback is that the calculation of the nADD depends on how precise the animals treated had the estimated standard live weight and were treated exactly with the recommended dosage. If the nADD and the nUDD differ the presumption that the veterinarian chose the wrong dosage would not be appropriate. Although it can be expected that for a large population, e.g., all animals within a country, the nUDD is similar to the nADD (because the estimated standard live weight should represent the average live weight of the treated animals), this will probably not be the case for results on an individual farm, e.g., because the average live weight of treated animals in this farm may differ from the estimated standard live weight. Furthermore, as production systems may vary considerably between countries, a systematic bias may be introduced by calculating nADDs based on European standard live weights for animal species/production types.

In our study we calculated the nADD according to the Danish procedure [22,30] and compared them to the nUDD as collected within our study. Regarding the estimated standard live weights we followed the Dutch calculation method [32], because the live weights used seemed to fit better to the German situation (expert opinion).

For piglets and calves most of the UDDs were above the recommended dose, while regarding sows, fattening pigs, dairy and beef cattle, most of the UDDs were lower than the respective ADD. Possibly the young animals treated had higher live weights than the estimated standard weight (underestimation of standard live weights results in underestimation of ADD) or they received higher dosages depending on the health status.

The frequent high percentage of records above the recommended dose concerning tetracyclines in almost all age groups (except sows and fattening pigs) can indicate evidence based adaption of dosages by the veterinarians. For some pharmaceuticals the dosages recommended by the manufacturer are below the dosages recommended in current literature, because they were authorized many years ago and the pathogen’s susceptibility changed since then.

In sows, the percentage of nUDD below the recommended dose was high compared to the other production types. It has to be suggested that the estimated standard live weight of 220 kg for sows is too high, because sows more often receive individual or parenteral treatments with usually precise dosages than other animal groups. Thus it seems to be more likely that the used dose UDD was correct, but the recommended dose ADD was calculated too high basing on the estimated live weight. Likewise fattening pigs that are treated with an antimicrobial are most probably lower in weight than 70.2 kg, and thus the percentage of nUDD below the recommended dose reached 49% in this study. It is noticeable that the percentage of records below the recommended dose was statistically significant lower than the respective nUDD for fattening pigs and sows as well as for dairy cattle. This means that treatments of numerous animals are more likely to be underdosed than records concerning few animals.

In calves, frequent underdosing was only observed for aminoglycosides, fluoroquinolones and fenicoles, possibly indicating higher live weights than the estimated 80 kg. The frequently used cephalosporins (often applied for udder diseases) for dairy cattle were applied below the recommended dose only in 36% of nUDD, and thus can be regarded as being within the recommended dose. Fluoroquinolones were used in dairy cattle below the recommended dose only in 22% of records, but in 56% of nUDD. This is a hint that treatment of single animals (e.g. treating udder diseases) are dosed within or above the recommended dose while group treatments (several animals per record) often are dosed below the recommended dose, although all treatments were administered parenterally.

To quote the use of antimicrobials knowledge of the medical indication is crucial. Respiratory and intestinal diseases are very common in livestock husbandry, because they are often caused by rather contagious pathogens which are frequently distributed within and between herds. The fact that the percentage of nUDD below the recommended dose was lower regarding enteric diseases than respiratory diseases may be related to the age of the animals: young animals often suffer from diarrhea, respiratory diseases also occur in older animals, e.g., fattening pigs at the end of their life. Therefore additional data concerning the treated animals are required in order to avoid the uncertainty of calculations of ADDs due to misestimation of live weights.

Respective infections are treated herdwise in order to treat all animals that are likely to be infected (metaphylaxis). In large herds (pigs and poultry), oral treatment via feed or water is the most common form of administration, because animals that still take up feed or water can be easily reached and individual treatment by injection would be stressful for them. The guidelines of antibiotic treatment and those for oral application of drugs to farm animals emphasize the prudent use of antibiotic treatment [4].

It was expected that parenteral treatment was dosed more precisely than oral treatment. This could be shown by comparing used and recommended doses. In cattle, a respective trend could be seen. 28% of records for parenteral treatment vs. 18% for oral treatment were within the recommended dose, although regarding the nUDD the percentage of applications within the recommended dose was higher for oral than for parenteral applications (18% and 25% of nUDD, respectively). 29% of parenteral applications (records) of cattle were above the recommended dose while doses in oral treatments were more often (52%) above the recommended doses. Underdosage of antimicrobials was detected in about 42% of all records from cattle, these results corresponding to the findings of other authors [25,26]. Ampicillin, spectinomycin and sulfonamides & trimethoprim seemed to be underdosed regularly, while tetracyclines were frequently used above the recommended dosage.

In pigs, the dosages used varied widely compared to the recommended dosage. 38% of records were below, 41% above and only 21% within the recommended dose. Results differed only slightly between age groups and administration routes. Nonetheless, the recommended dosage was within the 25%-quartile and the 75%-quartile of the used dosages for most of the antimicrobials. The fact that tetracyclines were used regularly above the recommended dosage in pigs and in cattle may be reflected in the fact that veterinarians prescribe higher doses due to previous treatment failures. As current practice does not lead to regular update of SPCs, new mechanisms should be established to ensure proper treatment recommendations on the SPCs. The newer antimicrobials: macrolides, fluoroquinolones and pleuromutilines were often applied within the recommended dosage.

Nonetheless, the evaluation of the true implications of these findings on the development of antimicrobial resistance is limited, because the assessment of prudent use requires detailed information that is not fully available from monitoring data. Thus, monitoring data can be used to observe trends in the dosage applied and to initiate a reassessment of the current dosage recommendation.

## Conclusions

Proper investigation of antibiotic use is challenging. By calculating and comparing UDDs and the respective ADD it is possible to estimate the impact of treatments regarding the risk for the selection of resistant bacteria. This approach can serve as a general overview of dosage patterns and may be able to reveal differences between countries or time periods. Nevertheless, it cannot be used to assess the dosage patterns of single treatments, because differences between UDD and ADD may be due to the animal live weight or the individual health conditions.

A longitudinal evaluation of antibiotics use in animals plays an important role in controlling resistance. Combining consumption data with those of the spread of resistant bacteria will help to bring resistance prevention and control a step forward.

## Competing interests

The authors declare that they have no competing interests.

## Authors’ contributions

MR, C H-G and YM carried out the feasibility study and recorded the data from the farmers and veterinarians. MR evaluated the pharmacological aspects of the study. C H-G analyzed the data from the veterinary practices, and YM the data from the farmers. RM and PH designed the study. PH prepared the study database, RM co-ordinated and supervised the statistical analyses. AK, WH and LK applied for grants, co-ordinated the study and were involved in drafting the manuscript. All authors read and approved the final manuscript.
